# Association between neurofibromatosis type 1 and cerebrovascular diseases in children: A systematic review

**DOI:** 10.1371/journal.pone.0241096

**Published:** 2021-01-04

**Authors:** Beatriz Barreto-Duarte, Fabiana H. Andrade-Gomes, María B. Arriaga, Mariana Araújo-Pereira, Juan Manuel Cubillos-Angulo, Bruno B. Andrade

**Affiliations:** 1 Laboratório de Inflamação e Biomarcadores, Instituto Gonçalo Moniz, Fundação Oswaldo Cruz, Salvador, Bahia, Brazil; 2 Multinational Organization Network Sponsoring Translational and Epidemiological Research (MONSTER) Initiative, Salvador, Bahia, Brazil; 3 Curso de Medicina, Universidade Salvador (UNIFACS), Salvador, Bahia, Brazil; 4 Programa de Pós-Graduação em Clínica Médica, Universidade Federal do Rio de Janeiro, Rio de Janeiro, Brazil; 5 Curso de Medicina, Centro Universitário Faculdade de Tecnologia e Ciências (UniFTC), Salvador, Bahia, Brazil; 6 Faculdade de Medicina, Universidade Federal da Bahia, Salvador, Bahia, Brazil; 7 Curso de Medicina, Escola Bahiana de Medicina e Saúde Pública (EBMSP), Salvador, Bahia, Brazil; Kaohsuing Medical University Hospital, TAIWAN

## Abstract

**Background:**

Neurofibromatosis type 1 (NF-1) is an autosomal dominant disease that affects one in every 3000 individuals. This disease can present a wide range of clinical manifestations, ranging from skin abnormalities to severe vascular damage. Although not commonly recognized in the context of NF-1, cerebrovascular disease (CVD), can be often present since childhood and diagnosed just later in life. When present, NF-1-associated CVD clinical manifestations may include headache, cognitive deficits and ultimately aneurysm rupture, causing death. Thus, CVD plays an important role in the clinical manifestations, disease severity and prognosis of patients with NF-1. This systematic review aims to summarize the body of evidence linking NF-1 and CVD in children.

**Methods:**

Two independent investigators performed a systematic review on the PubMed and EMBASE search platforms, using the following key terms: “neurofibromatosis type 1”, “Von Recklinghausen’s disease”, "children", "adolescents", "stroke", "Moyamoya disease", "vascular diseases", "cerebrovascular disorders", "aneurysm" and "congenital abnormalities". Studies focused on assessing the development of CVD in children with NF-1 were included.

**Results:**

Seven studies met the inclusion criteria. Twelve different clinical manifestations have been associated with cerebrovascular changes in children with NF-1; 44,5% of diagnosed patients were asymptomatic.

**Conclusion:**

The available evidence suggests that CVDs are related with the progression of NF-1, even in the absence of a clear clinical manifestation. In addition, improved prognosis was observed when imaging tests were performed to screen for cerebrovascular alterations early during the clinical investigation. Early diagnosis of CVD in NF-1 patients foster implementation of timely interventions, directly impacting clinical outcomes.

## Introduction

Neurofibromatosis type 1 (NF-1) is an autosomal dominant, chronic and progressive disease [1] with an incidence of approximately 1:3000 individuals, with almost half of the cases being related to a hereditary origin (familial NF) [2]. A mutation in the *NF1* gene, which is located in the chromosome 17 q11.2, results in the inability to synthesize the neurofibromin cytoplasmic protein. This latter protein acts as a modulator of the cell growth and differentiation beginning in the intrauterine life, is expressed by cells from the nervous system, endothelium and smooth muscle near blood vessels. The mutated protein and the related modifications of the cellular environment result in very high risk of cerebrovascular alterations. The gold-standard diagnosis of NF-1 is the molecular genetic testing, which is related to high cost and low availability in the public health system, especially in resource-limited regions [3]. For this reason, NF-1 diagnosis is frequently based on the presence of two or more criteria that encompass the main clinical manifestations of the disease and that were established by the National Institutes of Health (NIH) Consensus Development Conference [4]. NF-1 is constantly associated with vasculopathy and cerebrovascular abnormalities, with a pathophysiology that is still not completely understood [5–7]. Several NF-1 cases have been reported in children, but the incidence of cerebrovascular diseases (CVDs) associated with complications and the long-term impacts on NF-1 cases is still poorly recognized, which warrants more studies on the important topic [8].

There are multiple case reports in the literature of individuals with NF-1 that developed CVDs [9]. Following onset of CVDs in NF-1 patients, heterogeneous neurological manifestations and several complications have also been described [10]. These events frequently result in high morbidity and mortality in NF-1 patients [11]. The association of NF-1 with CVD is described to result in a wide range of pathologies, such as cerebral ischemia, aneurysms and *Moyamoya* Syndrome (MMS). The latter condition is characterized by progressive stenosis or occlusion of the internal carotid artery and its branches. For this reason, although several aspects regarding the natural history, determinants of symptoms, exact etiologic factors and clinical management of such syndrome are still unknown, routine vascular screening/assessment in NF-1 patients is indicated for early identification MMS [12]. Thus, CVDs in patients with NF-1 can actively be diagnosed since childhood. However, diagnosis is often delayed, as not all children with NF-1 undergo routine neuroimaging screening [13]. Furthermore, CVDs are still one of the major causes of death, with almost six million people annually [14], aside from being associated with significant morbidity worldwide, which makes them an important public health problem [15]. When CVD are detected early, proper clinical management is able to minimize its impact on adult life [11].

Therefore, detailed studies on the association between NF-1 and CVD are still needed [16]. In this present study, we performed a systematic review to summarize the body of evidence on the association between NF-1 and the development of CVD in children. Increasing knowledge in this field can drive development of more effective protocols to optimize diagnosis and therapy to reduce both mortality and the number of hospitalizations of these patients.

## Materials and methods

### Ethics statement

There were no patients directly involved in the research. The present study used publicly data from previously published studies to perform a systematic review. All information given to the research team was de-identified. Thus, the study was exempted from revision by the Institutional Review Board of the Instituto Gonçalo Moniz, Fundação Oswaldo Cruz, Salvador, Brazil, and did not require signed consent forms.

### Search strategy

A systematic review of NF-1 and its association with CVD in children aged between 0 to 18 years old was performed, in accordance with the recommendations of the Preferred Reporting Items for Systematic Reviews and Meta-Analyses (PRISMA) report (S1 Checklist). Two independent reviewers (BBD and FHAG) conducted the research in the following databases: PubMed and EMBASE.

**Fig 1:**
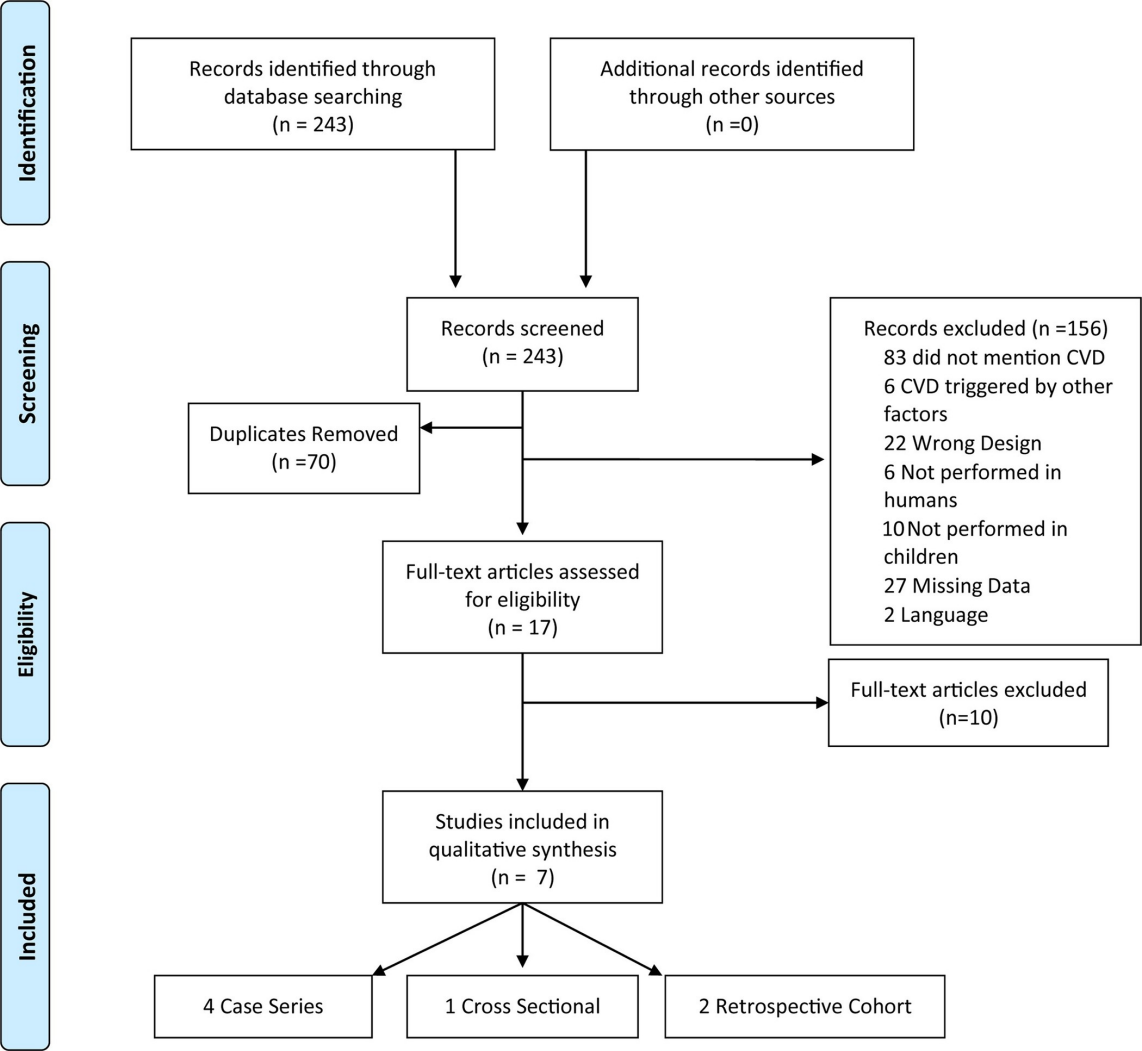
Flowchart of the study selection process study characteristics. Abbreviations: CVD: cerebrovascular disease.

The keywords used in the research were: "neurofibromatosis type 1", "Von Recklinghausen disease", "children", "adolescents", "stroke", "Moyamoya disease", "vascular diseases", "cerebrovascular disorders", "aneurysm", "congenital abnormalities". The search strategy used in PubMed and EMBASE medical literature databases using the consecutive terms in EMBASE: (('neurofibromatosis type 1':ti OR 'neurofibromatosis 1':ti OR nf1:ti OR 'nf 1':ti OR 'recklinghausen disease':ti) AND stroke OR (('neurofibromatosis type 1':ti OR 'neurofibromatosis 1':ti OR nf1:ti OR 'nf 1':ti OR 'Recklinghausen disease':ti) AND 'Moyamoya disease') OR (('neurofibromatosis type 1':ti OR 'neurofibromatosis 1':ti OR nf1:ti OR 'nf 1':ti OR 'Recklinghausen disease':ti) AND 'vascular disease') OR (('neurofibromatosis type 1':ti OR 'neurofibromatosis 1':ti OR nf1:ti OR 'nf 1':ti OR 'Recklinghausen disease':ti) AND vascular AND injury) OR (('neurofibromatosis type 1':ti OR 'neurofibromatosis 1':ti OR nf1:ti OR 'nf 1':ti OR 'recklinghausen disease':ti) AND 'cerebrovascular disease') OR (('neurofibromatosis type 1':ti OR 'neurofibromatosis 1':ti OR nf1:ti OR 'nf 1':ti OR 'Recklinghausen disease':ti) AND 'aneurysm') OR (('neurofibromatosis type 1':ti OR 'neurofibromatosis 1':ti OR nf1:ti OR 'nf 1':ti OR 'Recklinghausen disease':ti) AND vascular AND abnormalities) OR (('neurofibromatosis type1':ti OR 'neurofibromatosis 1':ti OR nf1:ti OR 'nf 1':ti OR 'Recklinghausen disease':ti) AND vascular AND malformation) OR (('neurofibromatosis type 1':ti OR 'neurofibromatosis 1':ti OR nf1:ti OR 'nf 1':ti OR 'Recklinghausen disease':ti) AND vasculopathy)) AND ('children' OR 'adolescent'). We used Mesh Term for PUBMED. All articles published in English were searched, without restriction regarding time or population. Initially, titles and abstracts were read by the reviewers (Fig 1) and systematic reviews, letters to the editor, case reports and comments were excluded. We did an additional manual search of the reference lists from selected articles to identify eligible publications that may not have appeared in our electronic search strategy. The search was conducted on May 15, 2020. This review was registered in the PROSPERO International Registry (registration number: CRD42020180942).

### Data extraction

The study inclusion criteria were: (1) cross-sectional, cohort or case-control studies; (2) studies focusing on NF-1 cerebrovascular disorders; (3) studies carried out with children (from 0 to 18 years old) and (4) studies in which the diagnostic criteria for NF-1 recommended by the NIH were used. Articles in a language other than English, duplicated or not yet published, as well as studies that were not about CVD in patients with NF-1 or that presented cerebrovascular events associated with trauma, neoplasia, radiation treatment or use of medications were excluded. The selection of the studies was divided into 4 steps: (1) reading of the titles, (2) reading of the article abstracts, (3) evaluation of the full articles selected from the previous step and inclusion of other studies present in the reference lists of the selected articles, (4) selection of the studies to include in the systematic review. Data extraction was performed independently by two authors (BBD and FHAG) and the discrepancies between the reviewers were resolved by consensus after discussion with more experienced authors (MBAG, BBA). A table for data extraction was built by each reviewer before writing the manuscript. The table included information on all relevant variables in each retrieved study. All data used in this review are available in the S1 Table.

### Data analysis

All studies addressed the following variables to assess the relationship between NF-1 and CVD in children: age at diagnosis of NF-1, age at the time of radiologic examination to identify CVD, findings of cerebrovascular alteration, affected vessels, method used for the diagnosis of NF-1, type of radiologic examination performed, clinical manifestations and indication of neurosurgery (Table 1).

**Table 1:** Summary and characteristics of the articles.

Study (author, year)	Country	Type of study	Sample size	Age of patients at NF1 diagnosis	Age of patients at image study	Cerebrovascular alteration	Affected vessel	Image study used	Diagnosis of NF1	Clinical manifestations	Neurosurgery
(Rosser et al., 2004)	United States of America	Case Series	Total (353); MRI (316); Vasculopathy (8)	Mean, 1.4 years	Mean, 7.3 years	Stenosis / occlusion, cerebral ischemia, ectasia, Moyamoya, aneurysm and hypoplasia	PCA, ICA and MCA	MRI, MRA and Conventional Angiography	NIH diagnostic criteria^a^	Hemiparesis (1), seizure (1) and asymptomatic (7)	3 patients (exploratory surgery, encephaloduroarteriosynangiosis and pial synangiosis)
(Cairns et al, 2008)	Australia	Case series	Total (698); MRI (144); Vasculopathy (7)	Ranged from 18 months to 5 years	Mean, 6.8 years	Hypoplasia, stenosis/occlusion, collateral vessels	ACA, MCA, PCoA, ICA and PCA	MRI, MRA and Conventional Angiography	NIH diagnostic criteria^a^	Asymptomatic (5), seizure (1), hemiparesis (1), paresthesia (1) and TIA (1)	1 patient (pial synangiosis)
(Rea et al, 2009)	Canada	Retrospective Cohort	Total (419); MRI (266); Arteriopathy (17)	Median, 2 years	Median, 5.2 years (7 children) Median, 5.3 years (10 children)	Stenosis / occlusion, aneurysm, Moyamoya	ICA, MCA and ACA	MRI, MRA, CT, angio-CT and Conventional Angiography	NIH diagnostic criteria^a^	Seizure (3), hemiparesis (1), speech delay (2), Bell's palsy (1), learning disability (4), paresthesia (1), weakness (2), hyperreflexia (2), ADD (1)), hyperphagia (1), episodic dysphasia (1), infantile spasms (1), hypertonia (1), clonus (1), ptosis (1), RAPD (1), hemiballismus (1), dystonia (1), aphasia (1), AIT (1) and fine motor delay (1).	6 patients (pial synangiosis)
(Ghosh et al, 2012)	United States of America	Case Series	Total (398); MRI (312); MRA (143); Vasculopathy (15)	Mean, 4.3 ± 3.5 years	Mean, 11.7 SD ± 7.3 years	Stenosis/occlusion, cerebral ischemia, Moyamoya	ICA, MCA, PCA and VA	MRI, MRA, angio-CT and Conventional Angiography	NIH diagnostic criteria^a^	Headache (5), seizure (2) and asymptomatic (7)	1 patient (encephaloduroarteriomyosynangiosis procedure)
(Kaas et al, 2012)	United States of America	Cross Sectional	Total (181); Neuroimaging (77); cerebral vasculopathy (12)	Median 8 years (n = 14)	Ranged from 3 to 13 years	Moyamoya, stenosis/occlusion, tortuosity, elongation, displacement, developmental venous anomaly	ACA, MCA, ICA, PCA and VA	MRI, MRA, Conventional Angiography	NIH diagnostic criteria^a^	Weakness (1), reduced responsiveness (1) and asymptomatic (10)	3 patients (2—revascularization surgery)
(Han et al, 2014)	China	Case Series	Total (6)	Median, 2.7 ± 2.1 years	Mean, 11.4 SD ± 8.3 years	Moyamoya, stenosis / occlusion, intracerebral hemorrhage	ICA, MCA and ACA	MRI, MRA, DSA, SPECT (Conventional digital subtraction angiography, Brain perfusion single-photon emission computerized tomography	NIH diagnostic criteria^a^	TIA (3), headache (1), intracerebral hemorrhage (1) and cerebral ischemia (1)	5 patients (Revascularization surgery)
(Santoro et al, 2017)	Italy/France	Retrospective cohort	Total (18)	Mean, 2.93 SD± 3.03 years	Mean, 7.43 SD ± 4.27 years	Stenosis/occlusion	MCA, ACA and PCA	MRI, MRA and DSA	NIH diagnostic criteria^a^	Hemiparesis (2), seizure (2), headache (6) and asymptomatic (8)	11 patients (revascularization surgery)

Note

^a^National Institutes of Health (NIH) Consensus Development Conference diagnostic criteria: consists of the presence of two or more of the following characteristics—six or more cafe au lait macules over 5 mm in greatest diameter in prepubertal individuals and over 15 mm in greatest diameter in post pubertal individuals; two or more neurofibromas of any type or one plexiform neurofibroma; freckling in the axillary or inguinal regions; optic glioma; two or more Lisch nodules (iris hamartomas); a distinctive osseous lesion such as sphenoid dysplasia or thinning of long bone cortex, with or without pseudarthrosis and a first-degree relative (parent, sibling, or offspring) with NF-1 by the above criteria.

**Abbreviations:** NF-1:Neurofibromatosis Type 1; PCA: posterior cerebral artery; MCA: middle cerebral artery; ACA: anterior cerebral artery; ICA: internal carotid artery; PCoA: posterior communicating artery; VA: vertebral artery; MRI: magnetic resonance imaging; MRA: magnetic resonance angiography; CA: conventional angiography; CT: computed tomography; angio-CT: computed tomography angiography; DSA: digital subtraction angiography; SPECT: Brain perfusion single-photon emission computerized tomography; TIA: transient ischemic attack; RAPD: DPAR: relative afferent pupillary defect; ADD: attention deficit disorder; SD: standard variation.

### Quality assessment

The methodological quality of the studies included in the meta-analysis was assessed using the Newcastle-Ottawa Scale (NOS) (Table 2) [17]. NOS scores of 0–3, 4–6 and 7–9, were considered to be low, moderate and high quality, respectively. The overall methodological quality was then summarized based on the observed quality trends.

**Table 2:** Quality assessment of studies included in the systematic review by Newcastle Ottawa Scale.

		Selection				Comparability		Outcome/Exposure			Overall Score^a^
Source	Newcastle Ottawa Scale	1	2	3	4	5A	5B	6	7	8	
(Kaas et al, 2012)	Cross-Sectional	0	*	*	**	N/A	N/A	**	0	N/A	6
(Santoro et al, 2017)	Retrospective Cohort	*	0	*	*	*	N/A	*	*	*	7
(Rea et al, 2009)	Retrospective Cohort	*	0	*	*	N/A	N/A	*	*	N/A	6

Note

*Indicates the score given to the study according to the NOS quality assessment scale.

Abbreviations: NA, not applicable

^a^ Determined by the total number of stars assigned to the study: 0–3 stars = poor; 4–5 stars = fair; 6–7 stars = good; 8–9/10 stars = excellent.

## Results

### Study characteristics and quality assessment

Initially, 243 studies were selected from the main database search. After detailed review, 70 duplicates were removed and 156 articles were excluded: 83 did not address CVD, 6 addressed CVD triggered by other factors, such as a particular medicine or neoplasms, 18 were case reports and 4 were letters to the editor (Fig 1). Subsequently, 6 other studies were excluded for not being performed in humans, 10 for not being performed in children, 27 were not found or published and 2 were in a language other than English (Polish and German) (Fig 1). Finally, 10 studies that did not provide sufficient clinical data on the pediatric population were excluded. The seven remaining studies, which described NF-1 associated with CVD in children, were included in this review. Fig 1 summarizes the article selection process.

**Fig 2:**
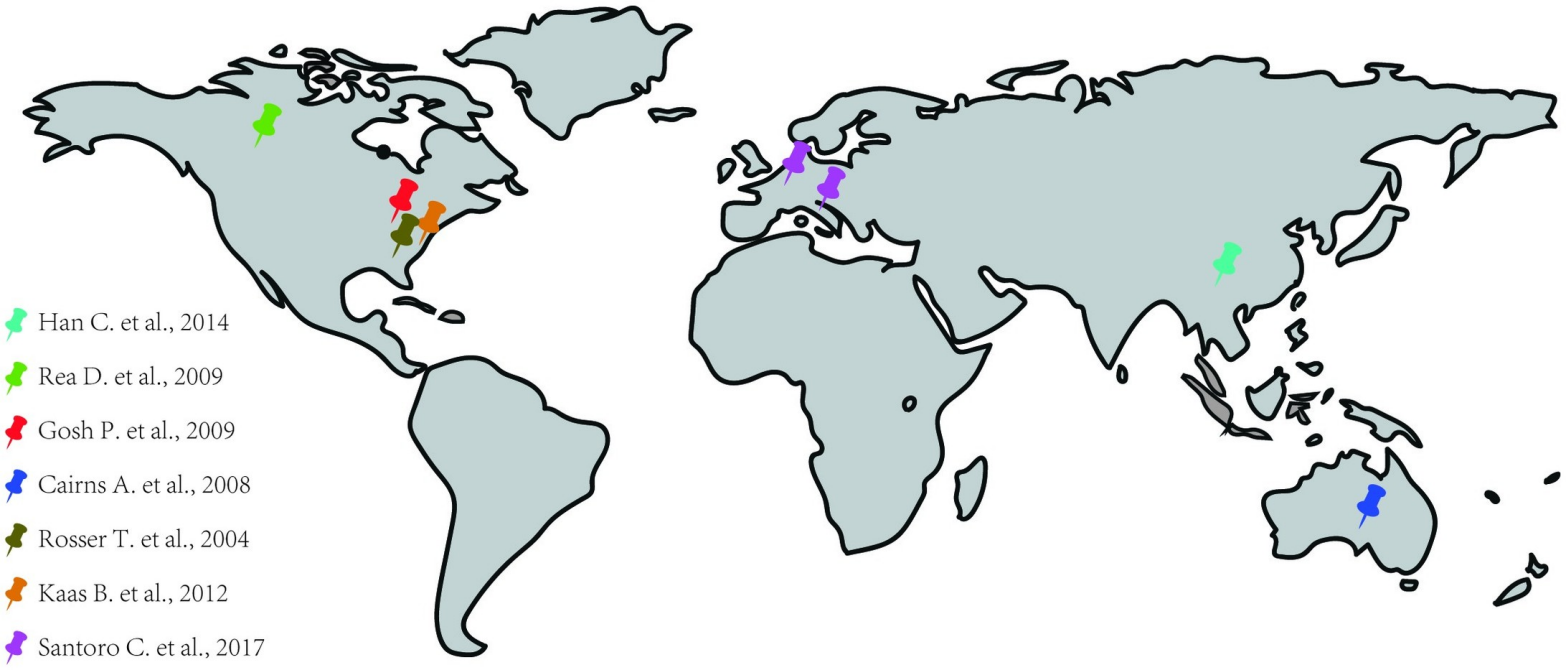
Distribution of studies used around the world.

Among the seven selected studies, 2 had a medical record review project (retrospective cohort), 1 was a cross-sectional study and 4 were case series. In all studies, the NF-1 diagnostic criterion used was that developed by the NIH. Moreover, the eligible studies (cohort and cross-sectional) for the evaluation of the Newcastle-Ottawa Scale (NOS) were of good quality presenting between 6–7 stars (Table 2). Such studies were performed in six different countries: 1 in Australia, 1 in Canada, 1 in China, 1 in Italy and France and 3 in different cities in the United States of America (Washington DC, Cleveland and Baltimore) (Fig 2).

### Cerebrovascular alterations were more frequent in the Willis polygon

**Fig 3:**
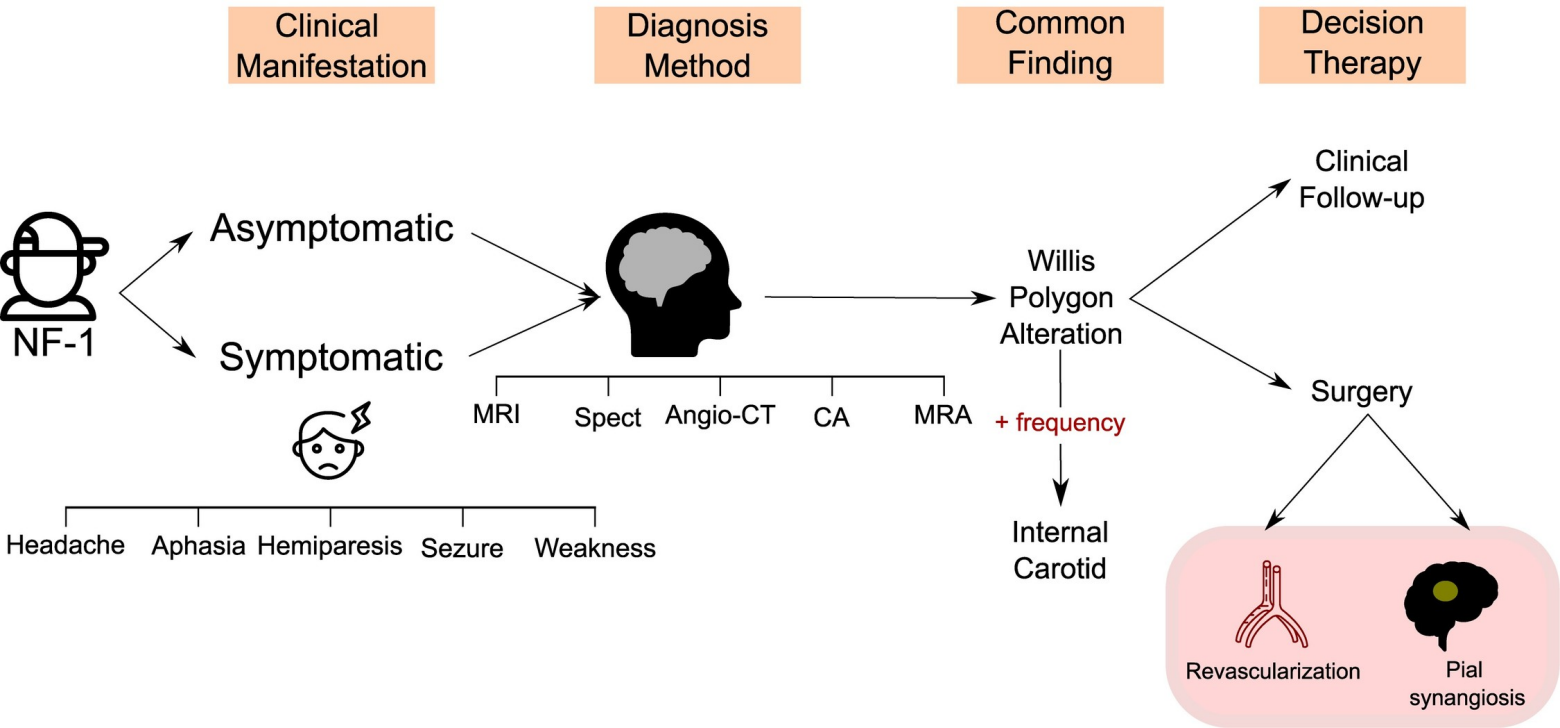
Flow diagram summarizing the main results of the systematic review. NF-1:Neurofibromatosis Type 1; MRI: magnetic resonance imaging; MRA: magnetic resonance angiography; CA: conventional angiography; CT: computed tomography; angio-CT: computed tomography angiography; SPECT: Brain perfusion single-photon emission computerized tomography.

In this review, we detected 12 types of cerebrovascular alterations which were significantly associated with NF-1 and / or disease progression in children (Table 1 and Table 3). In six of the seven studies, the internal carotid artery was the most commonly affected vessel: it has occurred in 75% of the patients reported by Rosser et al., in 80% in the study by Ghosh et al., and in 100% in the studies by Read et al., Kaas et al., Han et al. and Santoro et al. In the study by Cairns et al., the most frequently affected vessels were those from the Willis polygon (85,7% of patients) (Table 3 and Fig 3).

**Table 3:** Frequency of presentation of CVD in each study.

Study (author, year)	Number of patients with CVD	CVD (%)
(Rosser et al., 2004)	8	Stenosis/occlusion (50), Cerebral ischemia (37,5), Ectasia (12,5), Moyamoya (25), Aneurysm (12,5) and Hypoplasia (12,5)
(Cairns et al, 2008)	7	Hypoplasia (14,3), Stenosis/occlusion (100), Collateral vessels (28,6)
(Rea et al, 2009)	17	Stenosis /occlusion (94,1), Aneurysm (5,9), Moyamoya (76,5)
(Ghosh et al, 2012)	15	Stenosis/occlusion (100), Cerebral ischemia (6,7), Moyamoya (46,7)
(Kaas et al, 2012)	12	Moyamoya (41,7), Stenosis/occlusion (58,3), Tortuosity (25), Elongation (16,7), Displacement (8,3), Developmental venous anomaly (16,7)
(Han et al, 2014)	6	Moyamoya (83,3), Stenosis /occlusion (100), Intracerebral hemorrhage (16,7)
(Santoro et al, 2017)	18	Stenosis/occlusion (100)

**Note:** The percentages were calculated for each disease in the studies separately. Most patients have more than one disease.

Abbreviations: CVD: Cerebrovascular Disease.

### Radiologic investigation

The diagnosis of NF-1 was performed prior to brain radiologic exams in all studies, showing that many CVDs could have been previously diagnosed, which possibly would prevent complications or more invasive treatments. The most frequently performed imaging tests were Magnetic Resonance Angiography (MRA) and Magnetic Resonance Imaging (MRI), which were performed in 100% of the studies evaluated, followed by Conventional Angiography (CA) - 71.4%, Computed Tomography Angiography (Angio-CT) and Digital Subtraction Angiography DSA—28.5% each, and finally Brain Perfusion Single-photon Emission Computerized Tomography (SPECT) and Computed Tomography (CT) - 14.2% each (Table 2 and Fig 3).

### Heterogeneity of clinical manifestations and delays in diagnosis of CVD

NF-1 was shown to be closely associated with MMS in five studies and its presentation was related to worse outcomes and more intense clinical manifestations. In the study by Rea et al., It was possible to correlate NF-1 with the difficulty of developing cognitive functions such as reading, speaking and the ability to maintain focus/attention. Furthermore, in the study by Ghosh et al., five of the fifteen patients evaluated presented headache as the only clinical manifestation that could indicate CVD. Thus, this latter finding indicates that headache is possibly an important warning sign in the NF-1 population.

In general, the clinical manifestations reported by the different studies were very heterogeneous (Fig 3). Patients with a history of paresthesia, seizures, and even severe strokes were observed. However, it is important to note that until the time of CVD diagnosis, 44,5% of the patients were asymptomatic, which shows the importance of active screening from CVD in NIF-1 children. Furthermore, the type of surgery most commonly reported in the studies was *pial synangiosis* surgery (occurring in five out of seven studies) followed by revascularization surgery (three of seven studies) (Fig 3). In the study by Santoro et al., patients who underwent surgical treatment (50%) exhibited clinical stability.

## Discussion

NF-1 is a rare, autosomal dominant disease [18], which is strongly related with vascular malformations [19] and whose first symptoms commonly appear in childhood [20]. Such vascular alterations can occur in any body tissue/organ [8], but the greatest impact on morbidity and mortality occurs when vessels from the central nervous system (CNS) are affected [10]. Knowledge about the association between NF-1 and CVDs can contribute to a better understanding of the pathophysiology of NF-1 manifestations in the CNS. This information is also useful to guide development of novel approaches to prevent cerebrovascular events in children with NF-1. This systematic review demonstrated that the use of early radiologic methods is associated with early onset of interventions, being related with more frequently favorable outcomes, even in children who were still asymptomatic at the time of diagnosis.

Our search identified that all studies selected for the systematic analysis reported that arteries from the Willis polygon were affected [9, 11–13, 21, 22]. Among those vessels, the internal carotid artery was shown as the main site of vascular alteration between all the patients evaluated. Importantly, the branches generated by the internal carotid artery are the middle cerebral artery and anterior cerebral artery [23], predominantly responsible for motor and somatosensory deficits [24]. Thus, the clinical manifestations presented by the patients investigated may vary from a focal motor deficit to aphasia [13]. This diversity in clinical presentation has potential to delay diagnosis [25], because depending on the age of the child, verbal communication may still not be effective and motor skills may not be well-developed. Therefore, it is crucial that more studies are developed to assess the need for periodic screening in this young age group through brain imaging.

Of note, approximately 44.5% of the patients were asymptomatic at the time of the radiologic exams, which demonstrated vascular alterations and highlighted the need of early screening. At first glance, this fact counts against the NIH NF-1 management manuals since brain imaging is only indicated in the presence of symptoms. In addition, in the study by Ghosh et al., 33.4% of the patients evaluated presented headache as the only symptom, which can be justified by the fact that the most common vascular alteration reported in all studies was arterial stenosis/occlusion. Moreover, the comparison between the selected studies about children with NF-1 and cerebrovascular diseases revealed that the earlier the radiologic tests are performed, the better is the final clinical outcome due to immediate implementation of therapies, even when the children are still asymptomatic at the time of the examination. This scenario corroborates with the hypothesis that performing periodic and early examinations can directly reduce the morbidity and mortality and improve the quality of life of children with NF-1.

The systematic analysis of available literature allowed us to conclude that the neurological manifestations of NF-1 in pediatric patients are heterogeneous and difficult to diagnose clinically, and in most cases, it is necessary to perform a complementary radiologic test. In addition, when these manifestations are associated with MMS, the children usually develop a worse prognosis [22]. Notably, in 6 out of the 7 studies investigated here, MMS was described to directly relate with severity of CVD symptoms children in NF-1 children [12]. Moreover, the neurosurgery most frequently performed was the pial synangiosis surgery, which is widely used for the treatment of MMS. An important observation is that the surgical interventions performed were also more successful when associated with early radiologic tests, corroborating with the idea of early screening for identification of CNS vascular malformations. The radiologic tests most frequently reported by the studies were MRA and MRI, and there is still no consensus on which of those tests is more appropriate to be routinely performed or even with regard to the periodicity established for the examination.

Our study has important limitations. Some studies included here did not contain sufficient information about the pediatric groups, not allowing an adequate assessment. In addition, most studies were only descriptive, not performing statistical analysis of the data presented, which eliminated our capacity to perform a meta-analysis. It is also important to highlight the low availability and quality of the studies carried out about this theme, which present a significant variable number of patients and just superficial analysis of each individual patient, which hampered the ability to compare different patient groups. Moreover, due to the small amount number of publications, it was necessary to use the case series, that imposed an important limitation since we were unable to apply an adequate quality scale to the studies. Noteworthy, a large portion of the studies included in our analyses is from North America, with only six cases from China. Therefore, it is possible that the available evidence may be a subject of ethnic bias, highlighting the need for studies in other regions of the globe to trace a more generalizable characterization of NF-1 presentation in children. In addition, the available data on the chronology of the radiologic exams and the reason for the indication of one method among others are very scarce, hampering proper interpretation of these parameters. Regardless of these limitations, we believe that this systematic review adds to the current knowledge in the field by summarizing the most relevant aspects of the available literature on CVDs in NF-1 patients, which is a rare disease that affects 1 in every 3000 live births [26] and has major complications [10]. Our study suggest that future investigations should be carried out with an adequate number of patients and with a greater focus on the pediatric population.

The results of this systematic review show that the possible implementation of screening measures using radiologic methods has the potential to improve the early diagnosis of cerebrovascular alterations in children with NF-1, fostering timely optimized interventions. This could potentially reduce the number of deaths and sequelae observed when the diagnosis of vascular damage occurs at later stages of the disease. More robust and better-quality studies are needed to clarify the frequency of radiologic investigation and which exams should be recommended for each age group.

## Supporting information

S1 Checklist Prisma guideline checklist. (DOC)

S1 Table Raw data. Table Note: ^a^National Institutes of Health (NIH) Consensus Development Conference diagnostic criteria: consists of the presence of two or more of the following characteristics—six or more cafe au lait macules over 5 mm in greatest diameter in prepubertal individuals and over 15 mm in greatest diameter in post pubertal individuals; two or more neurofibromas of any type or one plexiform neurofibroma; freckling in the axillary or inguinal regions; optic glioma; two or more Lisch nodules (iris hamartomas); a distinctive osseous lesion such as sphenoid dysplasia or thinning of long bone cortex, with or without pseudarthrosis and a first-degree relative (parent, sibling, or offspring) with NF-1 by the above criteria. Abbreviations: NF-1:Neurofibromatosis Type 1; PCA: posterior cerebral artery; MCA: middle cerebral artery; ACA: anterior cerebral artery; ICA: internal carotid artery; PCoA: posterior communicating artery; VA: vertebral artery; MRI: magnetic resonance imaging; MRA: magnetic resonance angiography; CA: conventional angiography; CT: computed tomography; angio-CT: computed tomography angiography; DSA: digital subtraction angiography; SPECT: Brain perfusion single-photon emission computerized tomography; TIA: transient ischemic attack; RAPD: DPAR: relative afferent pupillary defect; ADD: attention deficit disorder; SD: standard variation.
(XLSX)
